# Successful delivery of viviparous lantern shark from an artificial uterus and the self-production of lantern shark luciferin

**DOI:** 10.1371/journal.pone.0291224

**Published:** 2023-09-27

**Authors:** Taketeru Tomita, Minoru Toda, Atsushi Kaneko, Kiyomi Murakumo, Kei Miyamoto, Keiichi Sato

**Affiliations:** 1 Okinawa Churashima Research Institute, Okinawa Churashima Foundation, Motobu, Okinawa, Japan; 2 Okinawa Churaumi Aquarium, Okinawa Churashima Foundation, Motobu, Okinawa, Japan; 3 Okinawa Churashima Foundation Veterinary Hospital, Okinawa Churashima Foundation, Motobu, Okinawa, Japan; Tanta University Faculty of Agriculture, EGYPT

## Abstract

Our recent success in the long-term maintenance of lantern shark embryos in artificial uterine systems has provided a novel option for the medical treatment of premature embryos for captive viviparous elasmobranchs. The remaining issue with this system is that the embryos cannot survive the abrupt change in the chemical environment from artificial uterine fluid (AUF) to seawater during delivery. To overcome this issue, the present study developed a new protocol for seawater adaptation, which is characterized by a long-term and stepwise shift from AUF to seawater prior to delivery. This protocol was employed successfully, and the specimen survived for more than seven months after delivery, the longest captive record of the species. During the experiment, we unexpectedly detected bioluminescence of the embryonic lantern shark in the artificial uterus. This observation indicates that lantern sharks can produce luciferin, a substance for bioluminescence. This contradicts the recent hypothesis that lantern sharks lack the ability to produce luciferin and use luciferin obtained from food sources.

## Introduction

Approximately 60% of shark species are viviparous [1]. Thus, the development of extra-uterine life support systems, or artificial uteri, provides a novel option for the medical treatment of premature-birth shark embryos in public aquaria, especially for the conservation of threatened species. Attempts for the maintenance of viviparous shark embryos in artificial environments have been made by some groups (Stephens Fisheries Institute [2]; joined team of Tokai University and Aquarium Fukushima, unpublished), although maintenance throughout the gestation period has not been achieved. Recently, we reported the long-term incubation of the embryonic slendertail lantern shark *Etmopterus molleri* [3]. This genus is well known to be able to emit blue–green light from the luminous organs distributed on the body, providing essential information on the evolution of bioluminescence in elasmobranch fishes (e.g., [4]). However, the difficulties in the captive maintenance of this genus have highly limited the *in vivo* experiment/observation to these animals. Our artificial uterine system is unique in the usage of specific incubation fluid (“artificial uterine fluid” or AUF) that has osmotic pressure and salinity similar to those of shark blood plasma. This fluid is expected to allow embryos to maintain their blood chemistry with minimal osmoregulatory effort. This method greatly extended the incubation period of the species from less than one week to five months and allowed the successful growth of embryos to the natural birth size.

One of the technical issues remaining in the research conducted by Tomita et al. [3] is that the embryos failed to adapt to changes in the chemical environment during artificial delivery. The chemical composition of AUF is significantly different from that of seawater, with a salinity of approximately half that of seawater and a high urea concentration. Thus, the embryo undergoes a large shift in its chemical environment through delivery. To prepare for this environmental shift, we periodically and repeatedly exposed embryos to seawater during the last two months of embryonic incubation [3]. This preadapting process mimics the mechanism observed in the uterus of some viviparous sharks, in which external seawater is periodically introduced into the uterus (“uterine flushing”; [5]). However, this preadapting protocol was unsuccessful, and all incubated specimens died during the process of seawater adaptation after delivery.

Here, we report second-round experiments of embryonic incubation of slendertail lantern sharks using an artificial uterus following Tomita et al. [3]. In these experiments, we applied a new preadapting protocol that allowed successful artificial delivery. During the experiments, we observed light emission in the incubated embryos, the biological significance of which is also discussed.

## Methods

### Acquisition of the embryos

Four females of slendertail lantern sharks were obtained by local fishers during hook and line fishing on November 20, 2021, in the sea near Okinawa Island, Japan. After being caught, the sharks were placed in a container with aerated seawater on the boat. However, these sharks died during the 3-h trip back to the port and were donated to the Okinawa Churaumi Aquarium (Okinawa, Japan) for scientific purposes. After ultrasound examination to confirm pregnancy, a total of 15 embryos were recovered from the uteri and introduced into the artificial uterus. In addition to these four females, two deceased females were obtained in the same manner on January 15, 2022, and 15 embryos were recovered and introduced into the artificial uterus.

### Experimental design

We duplicated the artificial uterine system developed by Tomita et al. [3], and used both the original and replicated systems in this study. The embryos were randomly placed in each artificial uterus. The members in each artificial uterus were flexibly changed according to the condition of the embryos and artificial uterus.

**Fig 1:**
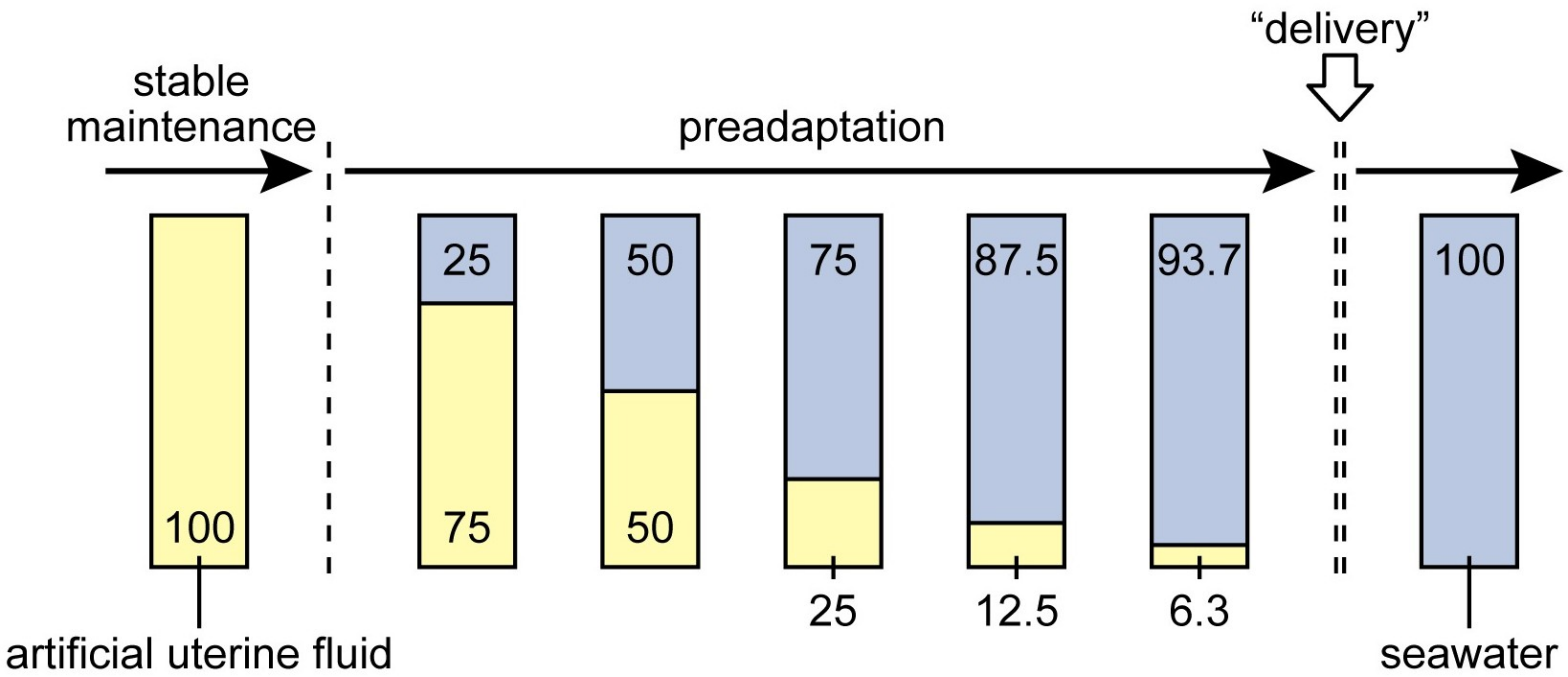
Overview of the incubation experiment of lantern shark embryos. The numbers shown in yellow and blue bars represent the volume percentages of artificial uterine fluid and seawater to make incubation fluid, respectively.

Embryonic incubation was performed in two steps: a stable maintenance period and a preadaptation period (Fig 1), in which the embryos were maintained in AUF in the same chemical composition as that described by Tomita et al. [3]. During the preadapting period, the incubation fluid was shifted from AUF to the full strength of seawater in a stepwise manner. During this period, the incubation fluid was made by mixing AUF and seawater in different proportions. Such seawater mixing has little effect on the osmotic pressure of the incubation fluid because the AUF has an osmotic pressure nearly equivalent to that of seawater (c.a., 1085 mmol/kg; [3]). The proportion of AUF in the incubation fluid decreased from 100 to 75, 50, 25, 12.5, and 6.3% in volume; conversely, that of seawater increased from 0 to 25, 50, 75, 87.5, and 93.7%. These proportional changes were completed within approximately two months. Each change in fluid composition was conducted in the following manner. First, the embryos were temporarily moved to a 70-L tank containing approximately 30 L of the original incubation fluid. The tank was immediately placed outside the artificial uterus. Second, 10 L of the incubation fluid was removed from the 70-L tank with a 500 mL-measuring cup, and 10 L of new fluid, with a different chemical composition, was introduced into the tank. This procedure was repeated approximately 10 times for 15 min, allowing the gradual shift of the embryonic environment into a new chemical composition. Third, the incubation fluid in the artificial uterus was removed with a vinyl hose and replaced with a new fluid. Finally, the embryos in the 70-L tank were moved to the artificial uterus. The entire process of this fluid exchange, from the movement of embryos outside of the artificial uterus to their recovery, was completed within approximately 2 h.

After the fluid composition in the artificial uterus reached the final state (i.e., a mixture of AUF and seawater at a volume ratio of 6.3 and 93.7%, respectively) through the preadaptation process, the composition remained stable until the external yolk sac of the embryos was fully retracted.

The fluid chemistry (sodium, urea, and ammonia) in the main chamber of the artificial uterus was monitored using an automated clinical chemistry analyzer (DRI-CHEM NX600, Fujifilm Co., Tokyo, Japan). Throughout the incubation period, water temperature was maintained at 12°C. The condition of the embryo was visually assessed twice a day (9:00 and 17:00) by observing the respiratory behavior (e.g., movement of gill flaps and spiracular valve) and the response to light stimuli (e.g., body undulation and changing body posture).

When the external yolk sac of the embryos completely retracted, we moved the embryos to a 500-L seawater tank and released them from the incubation jars. In analogy to a natural delivery, in which the embryo obtains great freedom in locomotion by releasing it from a spatially restricted environment, this process is defined as artificial delivery. The seawater tank was kept in the dark and the water temperature was maintained at 12°C. The pH of the water (8.2) was measured using a pH-indicator dye (thymol blue; Suzuken Co. Ltd., Tokyo, Japan). Ammonia and nitrate were not detected in the PACKTEST (WAK-NH_4_-4, WAK-NO_2_; Kyoritsu Chemical Check Laboratory, Tokyo, Japan).

The total length and body weight of the specimens were measured every month to monitor their growth after artificial delivery. Total length was measured from photographs using the built-in length measuring tool in ImageJ (US National Institutes of Health, Bethesda, MD, USA), and body weight was measured using the electronic balance EJ-6100B (A&B Co. Ltd., Tokyo, Japan). We did not weigh the specimens during the first two months after birth to avoid handling stress.

### Ethics statement

The maintenance and handling of live specimens in this study were conducted in strict accordance with the guidelines for animal experiments of the Okinawa Churashima Foundation, with the same consideration for animal care and welfare as that for “higher” vertebrates (reptiles, birds, and mammals). As the guidelines stipulate, the approval from the Institutional Animal Care and Use Committee of Okinawa Churashima Foundation, required for higher vertebrates, is waived for “lower” vertebrates, including fishes.

## Results

### Embryonic incubation

**Fig 2:**
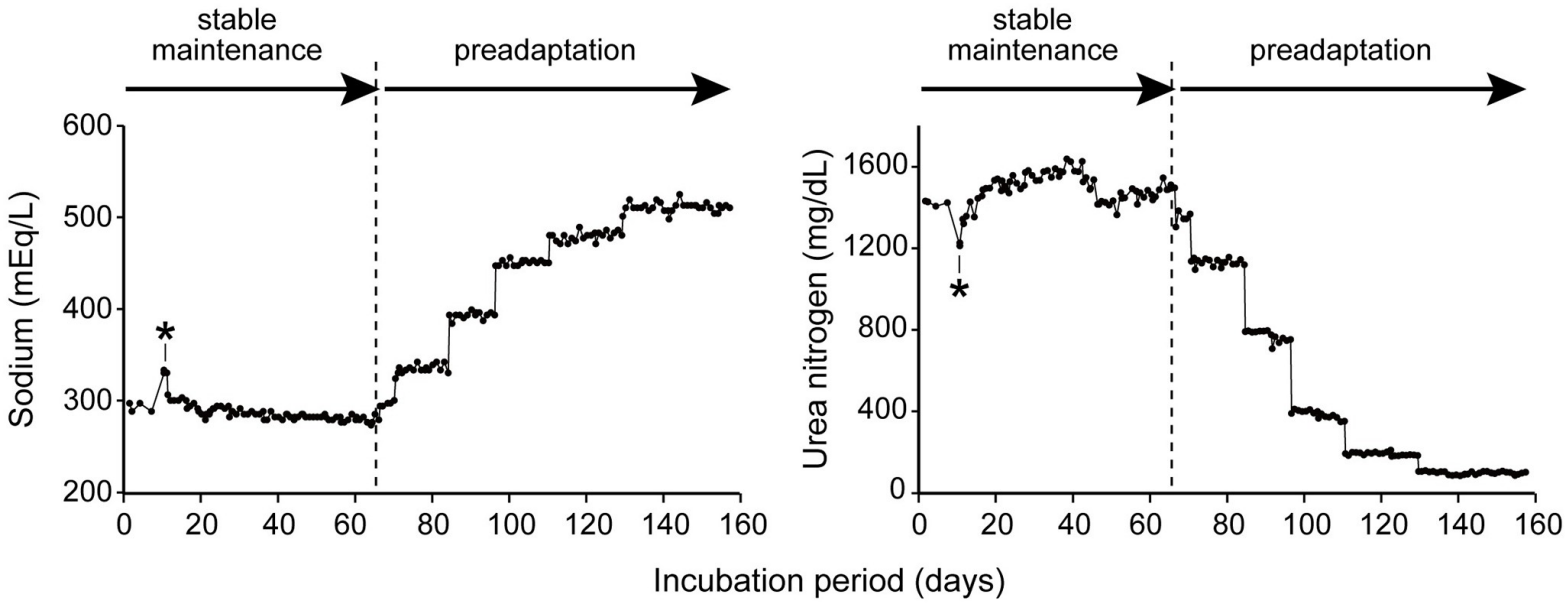
Temporal change in the chemical composition (sodium and urea) of the incubation fluid prior to artificial delivery. Earlier phase (stable maintenance) is characterized by the relatively stable composition in sodium and urea, whereas the later phase (preadapting process), by stepwise increase of sodium and decrease of urea composition toward the values of seawater. Note that the spike-like sodium increase (*) represents the seawater contamination to the artificial uterus.

Embryos were maintained in an artificial uterus for approximately four months. Throughout the incubation period, the total length increased from approximately 10 to 15 cm. The chemical composition of the incubation fluid is shown in Fig 2. Among the 30 embryos introduced into the artificial uterus, 20 embryos survived until the onset of the preadapting process. Embryonic deaths were caused by technical errors and occurred on November 29, 2021 and January 2, 2022; in the former case, two embryos died after the abrupt salinity increase caused by seawater contamination into the artificial uterus. In the latter case, three embryos died with probable oxygen deprivation caused by cessation of AUF supply to the incubation chamber. After two months of preadaptation, seven embryos survived. Most deaths during this preadapting period (16 out of 17 deaths) occurred in the very late phase of the preadapting process (the percentage of AUF in the incubation fluid was 12.5 and 6.3%). Among at least five of them, a yellowish mucus-like substance appeared on the skin surface before death, and a mass of bacteria was detected in this substance.

**Fig 3:**
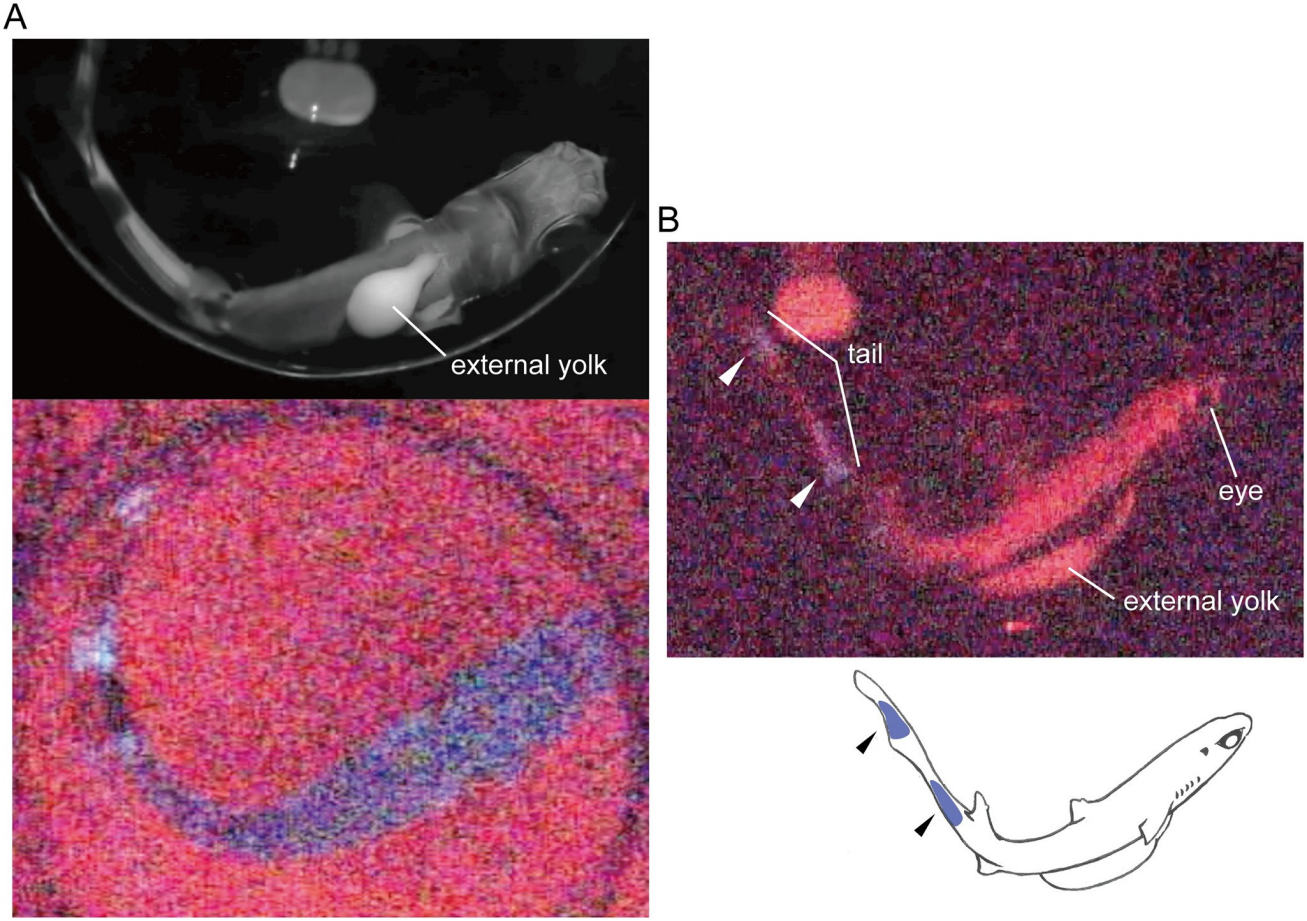
Bioluminescence of incubated embryos. A. Lower panel shows an image taken from high-sensitivity video footage (original video shown in S1 Video). Blue luminescence is seen on the ventral side from head to tail region of the incubated embryo. Upper panel shows an embryonic photo taken under stronger light conditions for better visualization of the body posture in the lower panel. Note that the specimens in the upper and middle panels represent different individuals and the upper panel was horizontally flipped for easier comparison. B. A lateral view of the embryo, showing the blue luminescence at the tail region (arrowheads).

During the preadaptation process, embryonic bioluminescence was unexpectedly recognized. Blue-green light was emitted from the ventral side of the trunk region (Fig 3 and S1 Video, taken with a high-sensitivity video camera (CANON/ME20F-SH [Canon Inc., Tokyo, Japan]). Bioluminescence was first detected on November 22, 2021 (day three of incubation), although its actual onset may have occurred earlier. Bioluminescence was observed on all four days on which video recordings were conducted (November 22, 2021; December 31, 2021; January 1, 2022; and February 23, 2022: day 3, 42, 43, and 96 of incubation, respectively). Up to 11 luminescent embryos could be identified if the potential duplicate counts between the videos were considered.

### Post-birth maintenance

Of the seven specimens that survived the preadaptation process, six were successfully born from the artificial uterus, but one, which was almost unresponsive to external stimuli prior to delivery, died on day 7 after delivery. During the early postnatal period, the specimens generally stayed at the bottom of the tank but swam actively in response to light stimuli. On day 3 after delivery, the specimens ate 0.1 g of fish paste, which was prepared by mincing the entire body of chub mackerel (*Scomber japonicus*) after removing its digestive tract, mixing with small amounts of minced sakura shrimp (*Lucensosergia lucens*), and then straining through gauze. These food items are available at the Okinawa Churaumi Aquarium and are often used for sustaining deep-water fishes. Another advantage of using these two items is that the mixing process increases the adhesiveness of the paste, enabling it to remain securely affixed to the feeding stick during feeding. Thereafter, the specimens were fed every four days, gradually increasing the amount to 0.8 g as they grew (S2 Video).

**Fig 4:**
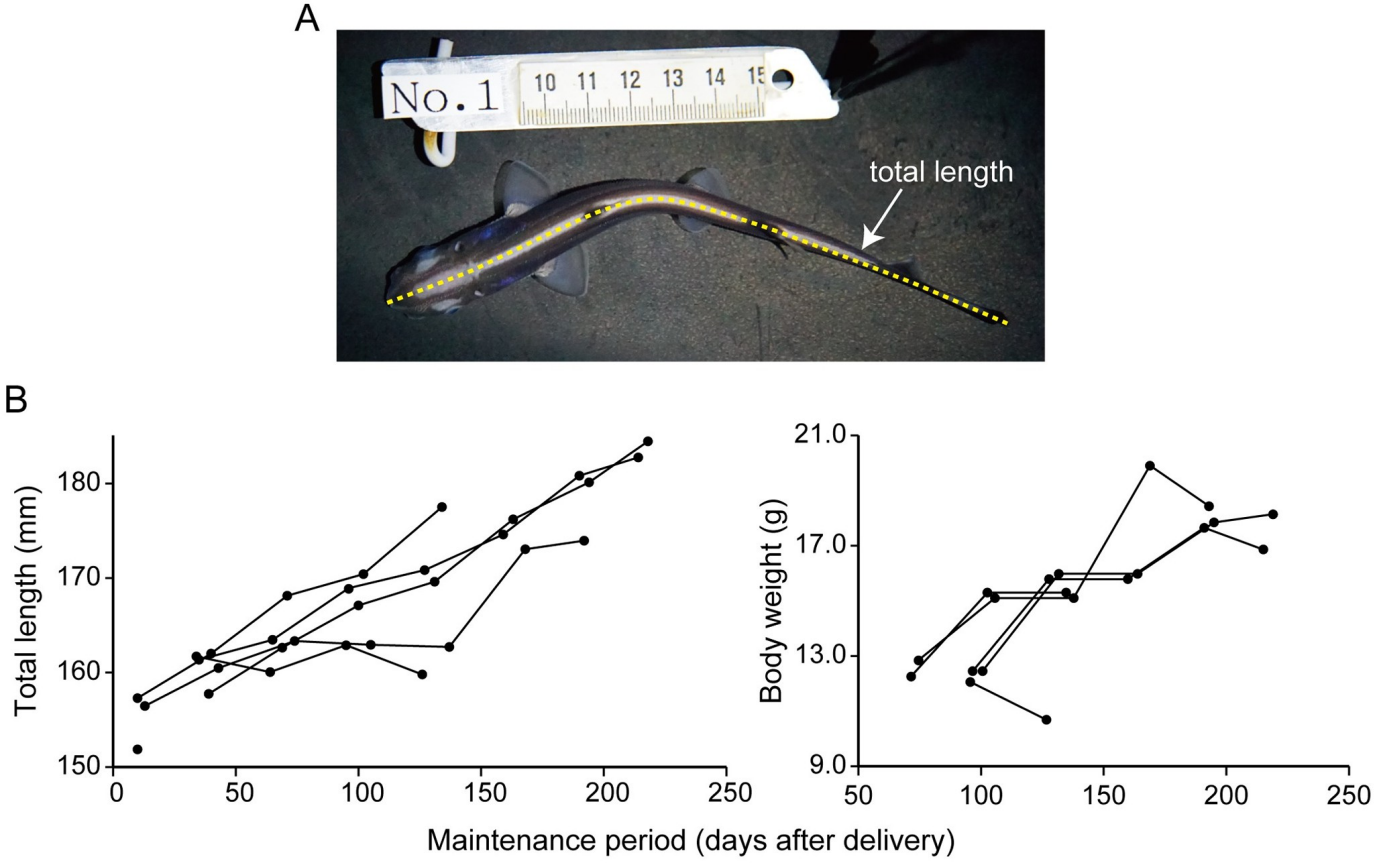
Growth process after artificial delivery. A. Photograph of postnatal shark showing the measurement of total length. B. Change in total length and body weight of six juveniles after delivery.

During maintenance in the seawater tank, the specimens exhibited increased locomotion, and abrasions in the snout and nasal regions became evident. In an attempt to rectify this situation, the specimens were transferred to a larger seawater tank (1,000 L); however, notable healing was not observed, and the specimens eventually died. Ciliate infection was observed in four specimens when they died. The average post-delivery maintenance period of the six specimens was 163.2 days (c.d. = ±77.1 days), with a maximum of 226 days. The maximum total maintenance period, including embryonic and post-delivery maintenance periods, was 357 days. Body size increased from approximately 16 to 18 cm in total length and from approximately 12.5 to 18 g during the last five months (Fig 4).

## Discussion

The preadapting method employed in this study is characterized by a long-term and stepwise shift of the embryonic environment from AUF to seawater in an artificial uterus. The survival of lantern sharks after delivery for more than seven months indicates that this preadaptation protocol worked successfully. A total of 357 days of captivity greatly extends the previous record of 184 days in Tomita et al. [3] and is the longest period for this species. Despite such improvements, there remains a technical issue of relatively high mortality in the final stages of the preadaptation process, with at least five of these deaths presumably resulting from bacterial infection. During the final stage of the preadaptation phase, the embryo must expend more energy on osmoregulation because of the greater difference in salinity between the embryonic plasma and the environmental fluid. The increased energy demand for osmoregulation may have reduced the energy budget for physiological functions other than osmoregulation, including the immune system. If so, the final phase of the preadaptation period should provide a high risk for bacterial infection, and a clean environment during this period is especially important. An environmental parameter not considered in this study is pH. A study of the uterine fluid of viviparous *Squalus acanthias* revealed high lactate concentrations, resulting in a lower uterine pH during pregnancy [6]. Although the physiological significance of low uterine pH remains under discussion (e.g., ammonia removal [6]), we hypothesize that the low pH level contributes to the prevention of bacterial growth in the uterus. If our hypothesis is true, mimicking a low-pH environment in an artificial uterus may decrease the risk of bacterial infection in the embryos.

In addition to seawater adaptation, successful initiation of oral feeding is also an important factor for juvenile survival. We encountered some cases in which newborn juveniles of captive viviparous sharks (i.e., *Nebrius ferrugineus* and *Orectolobus japonicus*) died after the first food intake at the Okinawa Churaumi Aquarium (MT, unpublished data). These deaths were presumably caused by inadequate stomach activity during the early postnatal period. It is possible that the stomachs of newborn juveniles are not fully functional, because elasmobranch embryos are nourished primarily from the yolk in the intestine, and stomach activation begins in the latest stage of gestation [7]. In the present study, we fed fish paste to pups of the slendertail lantern sharks with the aim of minimizing digestive stress to the stomach. After feeding, greenish feces were observed in the tank, suggesting that normal digestion occurred. This is in contrast to the case of [3], in which pups vomited stomach contents on the day after being fed small pieces of fish meat.

The present study provides novel insights into the source of lantern shark luciferin, a substance used for light emission. Lantern sharks have been hypothesized to use self-produced luciferin (e.g., [8]). Recently, Mizuno et al. provided another hypothesis regarding the source of the lantern shark luciferin [9]. The authors claimed that the lantern sharks do not have the ability to produce luciferin but “steal” it from prey. This hypothesis is based on the observation that coelenterazine, a luciferin from luminous copepods, is found in the digestive tract of the lantern shark. This hypothesis, however, contradicts the previous observation that coelenterazine is not detected in the luminous organ of lantern sharks [10], and thus the “luciferin-stealing” hypotheses remains controversial. The light emission observed in our incubated embryos indicates that they can produce light without any oral intake of coelenterazine, which supports the traditional notion that lantern sharks use self-produced luciferin. Such embryonic bioluminescence was also reported in the embryo of the velvet belly lantern shark (*Etmopterus spinax*) shortly after recovery from the mother [11]. However, it should be noted that our observation does not completely reject the “luciferin-stealing” hypothesis, because it is still possible that maternal coelenterazine, which was originally acquired from prey, is transferred to eggs or early term embryos. Further work should be performed to clarify this issue.

## Supporting information

S1 Video High-sensitivity video footage of lantern shark embryos maintained in artificial uterine fluid. Blue bioluminescence is clearly visible when the embryo rotated its body and turned its ventral side upward.
(MP4)

S2 Video Video footage showing postnatal lantern sharks feeding on fish paste. (MP4)
